# Supplementary material for: Natural Product-like Fragments Unlock Novel Chemotypes for a Kinase Target—Exploring Options beyond the Flatland
Source: J Chem Inf Model. 2025 Dec 22;66(4):2249–67. doi: 10.1021/acs.jcim.5c01952 (PMC12933889; doi:10.1021/acs.jcim.5c01952)
Supplement: Supplementary file 5 [file ci5c01952_si_005.pdf]

Supporting Information

Natural Product-Like Fragments Unlock Novel Chemotypes for a Kinase Target – Exploring Options beyond the Flatland

Anna Santura,[†] Janis Müller,[‡] Madita Wolter,[‡] Ina-Charlotte Tutzschky,[†] Moritz Ruf,[‡] Alexander Metz,[¶,§] Anna Sandner,[¶] Stefan Merkl,[¶] Gerhard Klebe,[¶] Serghei Glinca,[*,‡] and Paul Czodrowski[*,†]

†*Johannes Gutenberg University Mainz, Department of Chemistry, Duesbergweg 10–14, 55128 Mainz, Germany*

‡*CrystalsFirst GmbH, Marbacher Weg 6, 35037 Marburg, Germany*

¶*Philipps-University Marburg, Department of Pharmaceutical Chemistry, Marbacher Weg 6, 35037 Marburg, Germany*

§*Current address: UCB Biopharma SRL, Braine-l'Alleud, Belgium*

E-mail: serghei.glinca@crystalsfirst.com; czodpaul@uni-mainz.de

Contents

Binding Modes of Fragment Hits . . . . SI 2

Prior Knowledge on the Fragments and their Parents from Database Mining . . . SI 4

Allosteric Pocket Occupation . . . . SI 7

    F189 Binds at Peripheral Site E . . . . SI 9

Chemical Space Analysis . . . . SI 11

    Bemis-Murcko Scaffolds . . . . SI 11

    Chemotypes . . . . SI 12

    Molecular Descriptors . . . . SI 20

Binding Modes of Fragment Hits

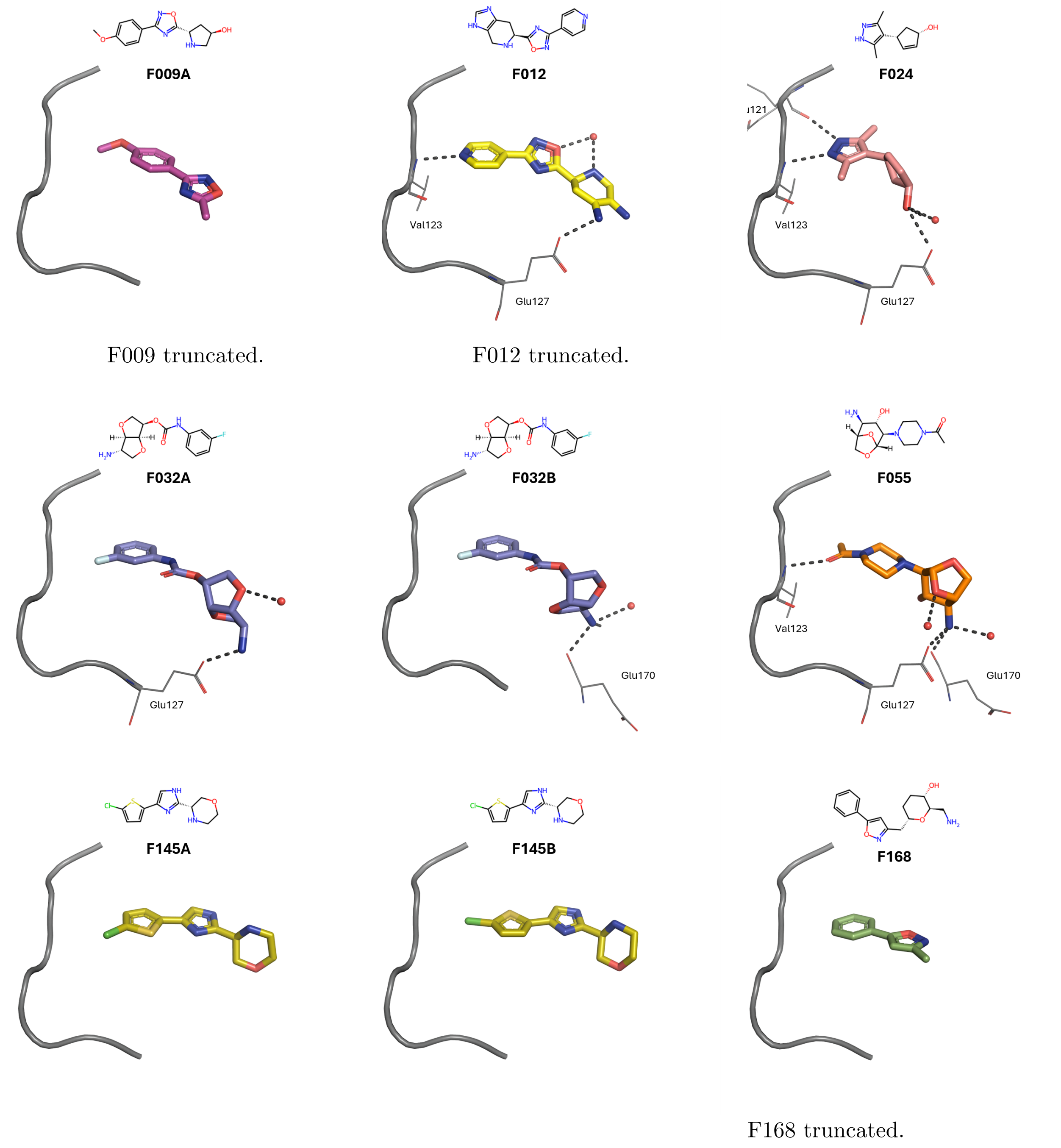

Figure S1: Binding modes of fragments within PKA's ATP pocket. If multiple conformations of the fragments were resolved, we show each individually (A/B). Fragments are shown as sticks, amino acid residues involved in polar interactions as lines, waters involved in polar interactions as red spheres, and polar interactions as dashed lines. Additionally, the kinase hinge and linker (residues 120 − 127) are shown in cartoon representation. For clarity, only amino acid residues involved in polar interactions are labeled. The assigned molecules were truncated to the regions with well-defined electron density at $\sigma = 1$ to minimize model bias.

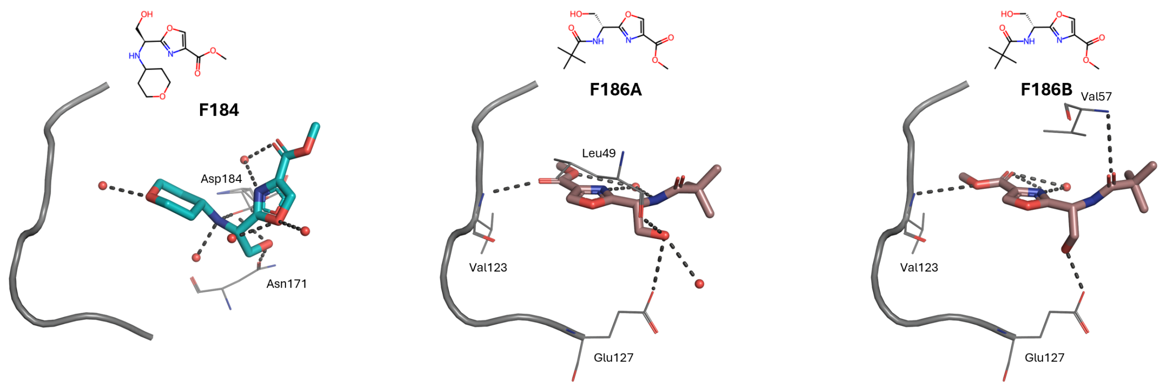

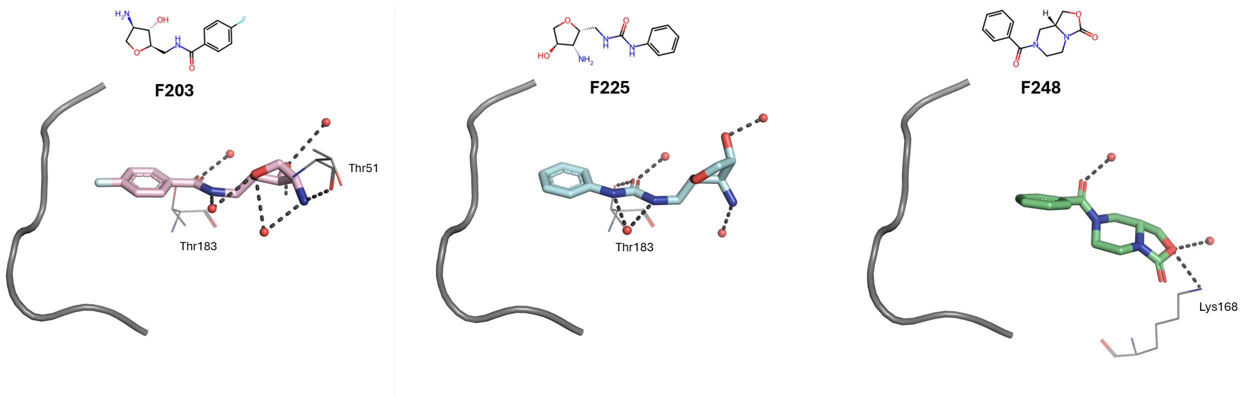

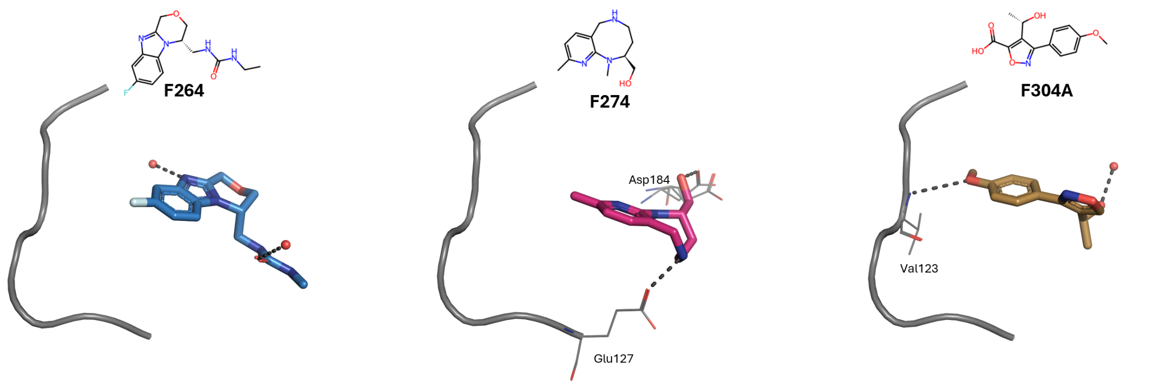

F264 truncated.

F304 truncated.

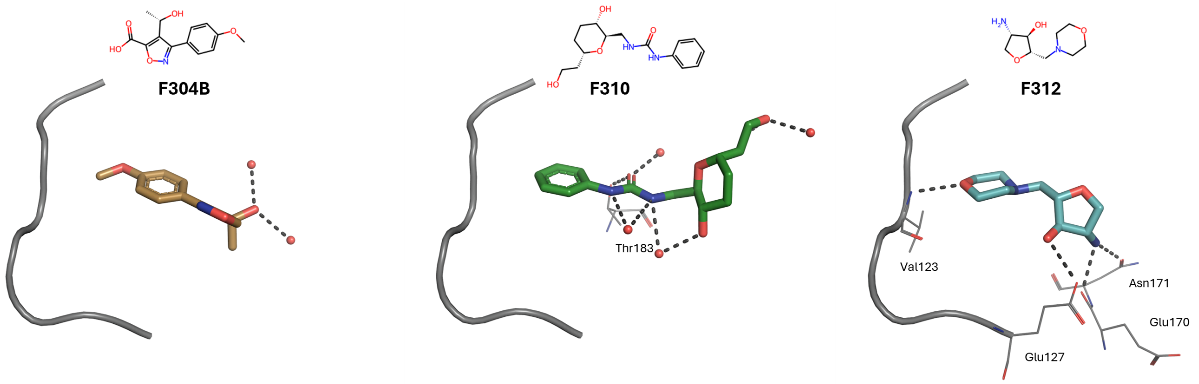

F304 truncated.

Fig. S1: Continued.

Prior Knowledge on the Fragments and their (Natural Product) Parents from Database Mining

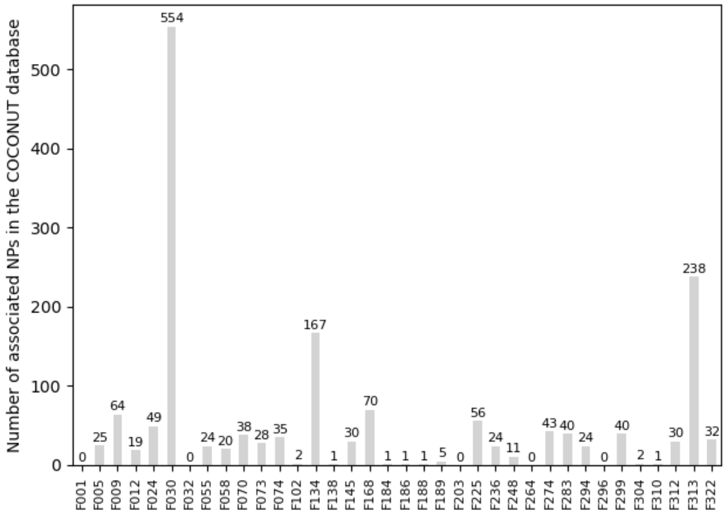

Figure S2: Number of Natural Products (NPs) from the COCONUT database, comprising our fragment hits as a substructure. Unsurprisingly, all the non-fluorinated fragments,i.e. all fragments except F001, F032, F203, F264 and F296, were found to possess an entry in the COCONUT database themselves, as the latter sources molecules from the subset of NPs in the ZINC15 database, among others, which in turn integrates the AnalytiCon Discovery libraries.

No NP was found in the COCONUT for F001, F032, F203, F264, and F296, all of which are fluorinated. According to the COCONUT curators, many of such compounds have been removed from their database, as there is a lack of conclusive evidence supporting their classification as NPs.Unsurprisingly, all the non-fluorinated fragments were found to possess an entry in the COCONUT database themselves, as the latter contains molecules from the subset of NPs in the ZINC15 database, among others, which in turn integrates the AnalytiCon Discovery libraries.

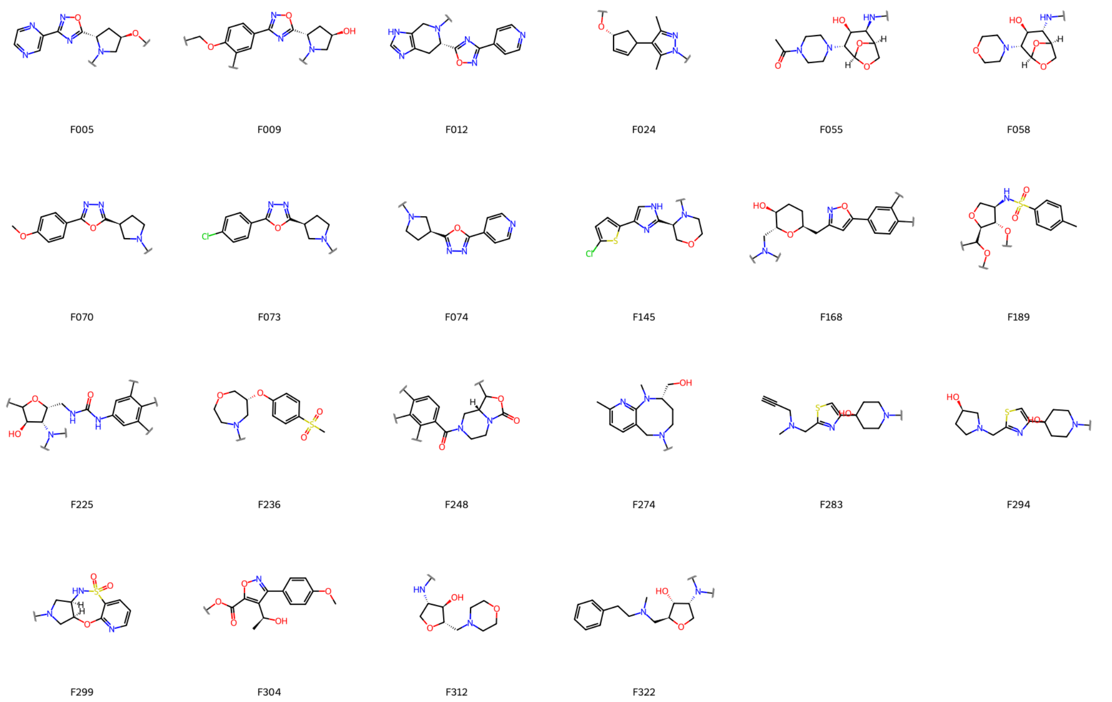

Figure S3: Growth vectors in the fragments, as deduced from the derivatization patterns of the associated natural products, indicated by a squiggled line perpendicular to a bond.

Glycogen synthase kinase-3β

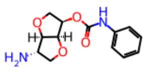

F030

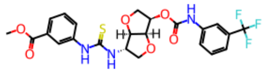

CHEMBL4791344
IC50 > 50.0 uM

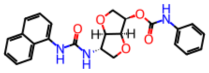

CHEMBL4791130
IC50 > 50.0 uM

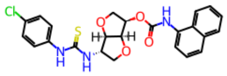

CHEMBL4778874
IC50 > 50.0 uM

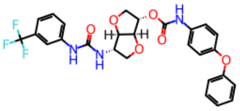

CHEMBL4761344
IC50 > 50.0 uM

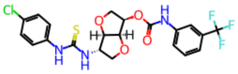

CHEMBL4758361
IC50 > 50.0 uM

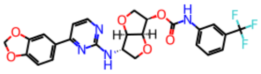

CHEMBL4751957
IC50 = 1.08 uM

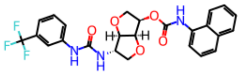

CHEMBL4741367
IC50 > 50.0 uM

mTOR

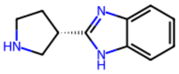

F134

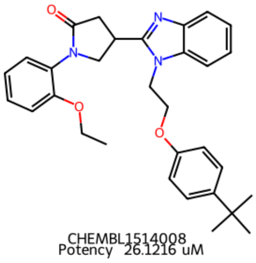

CHEMBL1514008
Potency 26.1216 uM

Figure S4: ChEMBL Molecules comprising a fragment hit as a substructure and their reported bioactivity towards a kinase. Molecules CHEMBL4791130 and CHEMBL4778874 were also found in the COCONUT-based substructure search (COCONUT-IDs = CNP0251686 and CNP0357796).

Allosteric Pocket Occupation

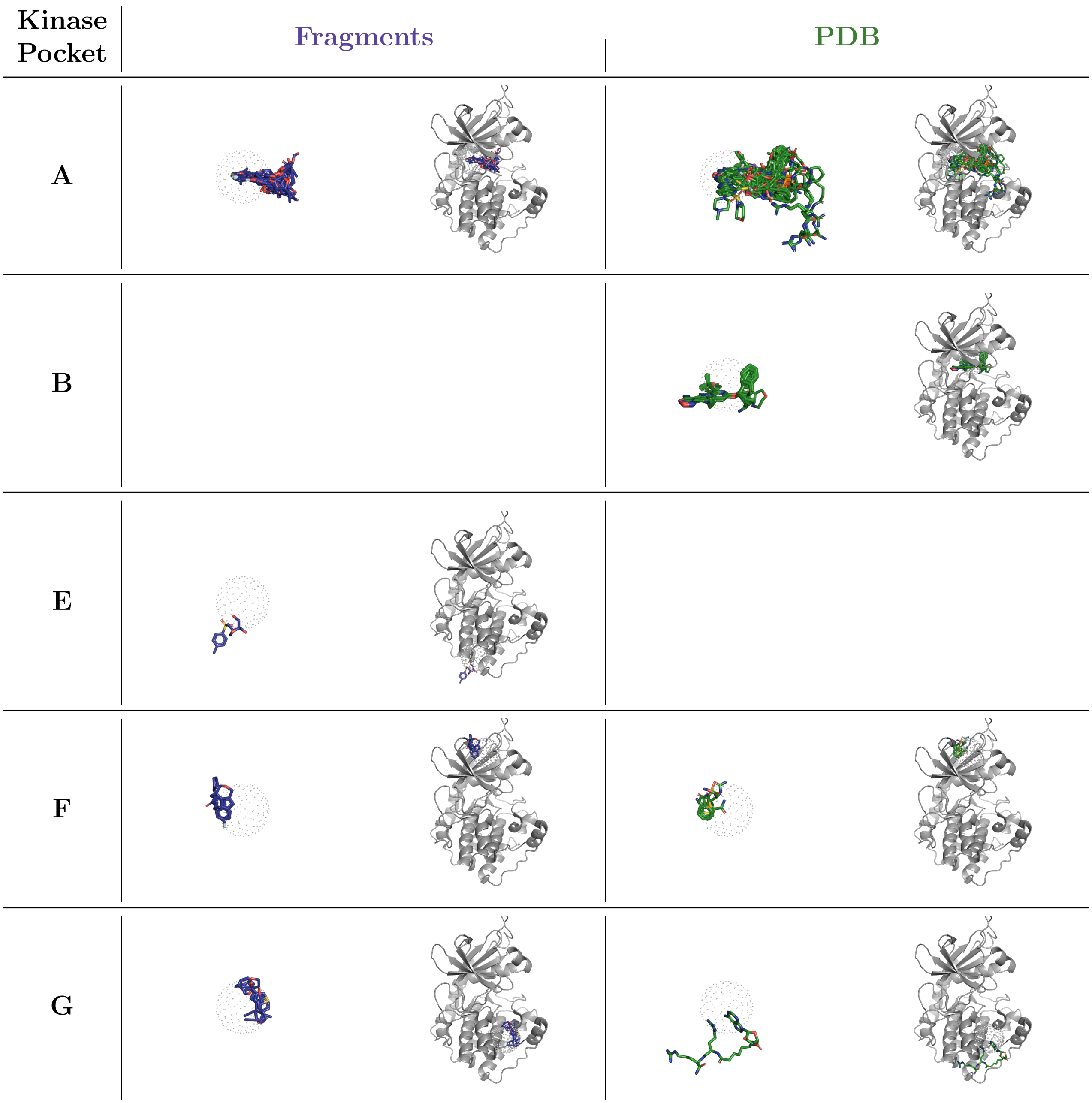

Figure S5: Allosteric pocket occupation by PKA ligands from the PDB and our fragment hits (stick representation with green and blue carbons, respectively). The pocket occupation of a ligand/fragment was defined as 'being located in a 5 Å radius (visualized as dots) of an added and well-considered pseudo atom' which in turn was placed in the center of mass of all ligands binding with this pocket as listed by Xerxa et al.. Thus, the position of ligands occupying the same pocket can be slightly shifted. For reference, the PKA structure (PDB-ID: 3FJQ) is shown in ribbon representation.

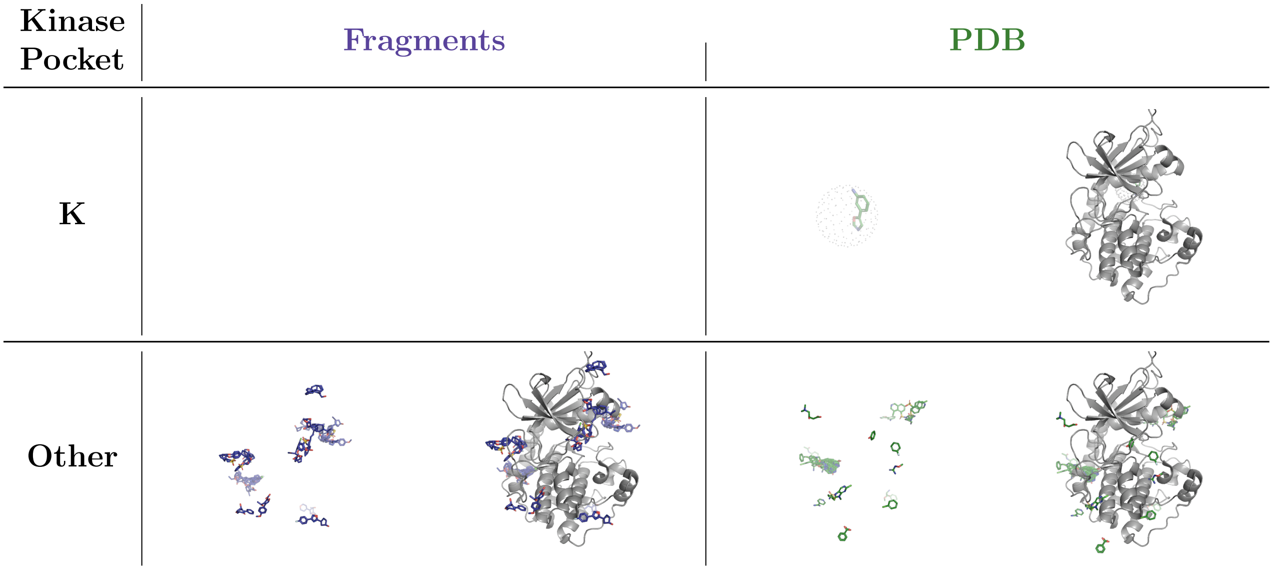

Figure S5.: Continued.

F189 Binds at Peripheral Site E

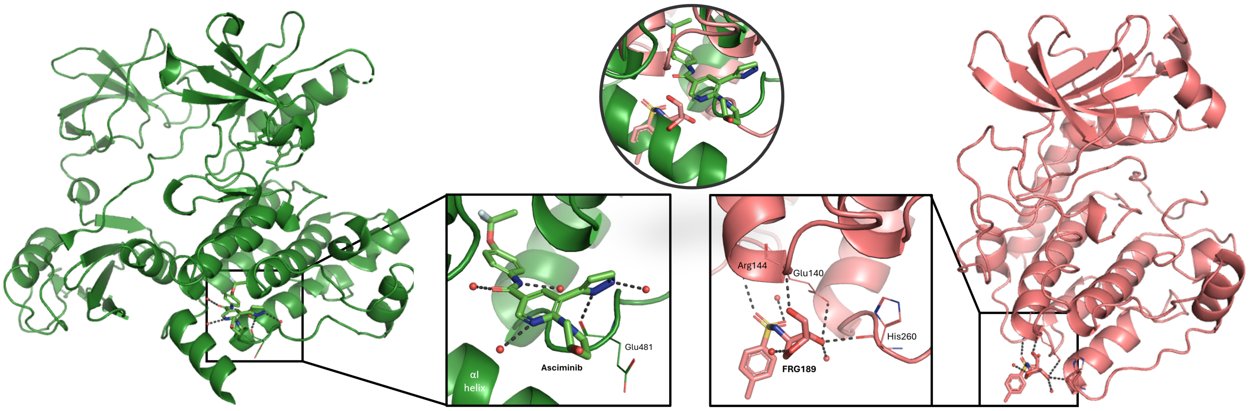

Figure S6: Comparison of binding modes for asciminib and F189. Ligands are shown as sticks, both kinases are shown in cartoon representation, hydrogen bonds are visualized as dashed black lines. Moreover, in the insets, amino acid residues involved in polar interactions are depicted as lines and water molecules as red spheres. For clarity, we show both complex structures and binding modes independently, but in the same orientation. **Left:** Asciminib bound to BCR-ABL1 (PDB-ID: 5MO4). The C-terminal αI-helix of tyrosine kinases such as ABL1 exhibits significant conformational flexibility and borders the allosteric pocket E in its kinked conformation,[56] which is not the case for PKA. **Right:** F189 bound to PKA. **Middle:** In addition, we show the superimposition of asciminib bound to BCR-ABL1 and F189 bound to PKA in the circular inset. Remember, that the pocket occupation of a ligand/fragment was defined as 'being located in a 5 Å radius of an added and well-considered pseudo atom' which in turn was placed in the center of mass of all ligands binding with this pocket as listed by Xerxa et al. (section Analysis of Ligand-Bound Protein Kinase A Crystal Structures). Thus, the position of ligands occupying the same pocket can be slightly shifted.

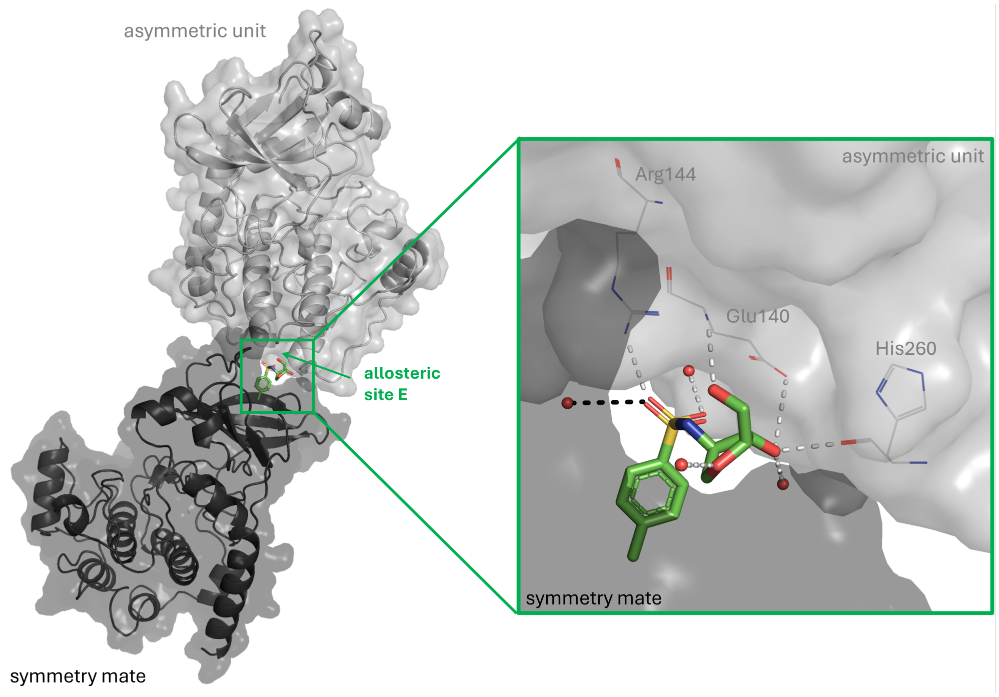

Figure S7: Hydrogen bonding interactions of F189 (stick representation with green carbons) within the asymmetric unit and with the neighboring symmetry mate in the crystal lattice (surface/cartoon representation, light gray and black, respectively). Hydrogen bonds are visualized as dashed white and black lines, respectively; the protein residues and water molecules involved in them are displayed in line representation or as spheres.

Chemical Space Analysis

Bemis-Murcko Scaffolds

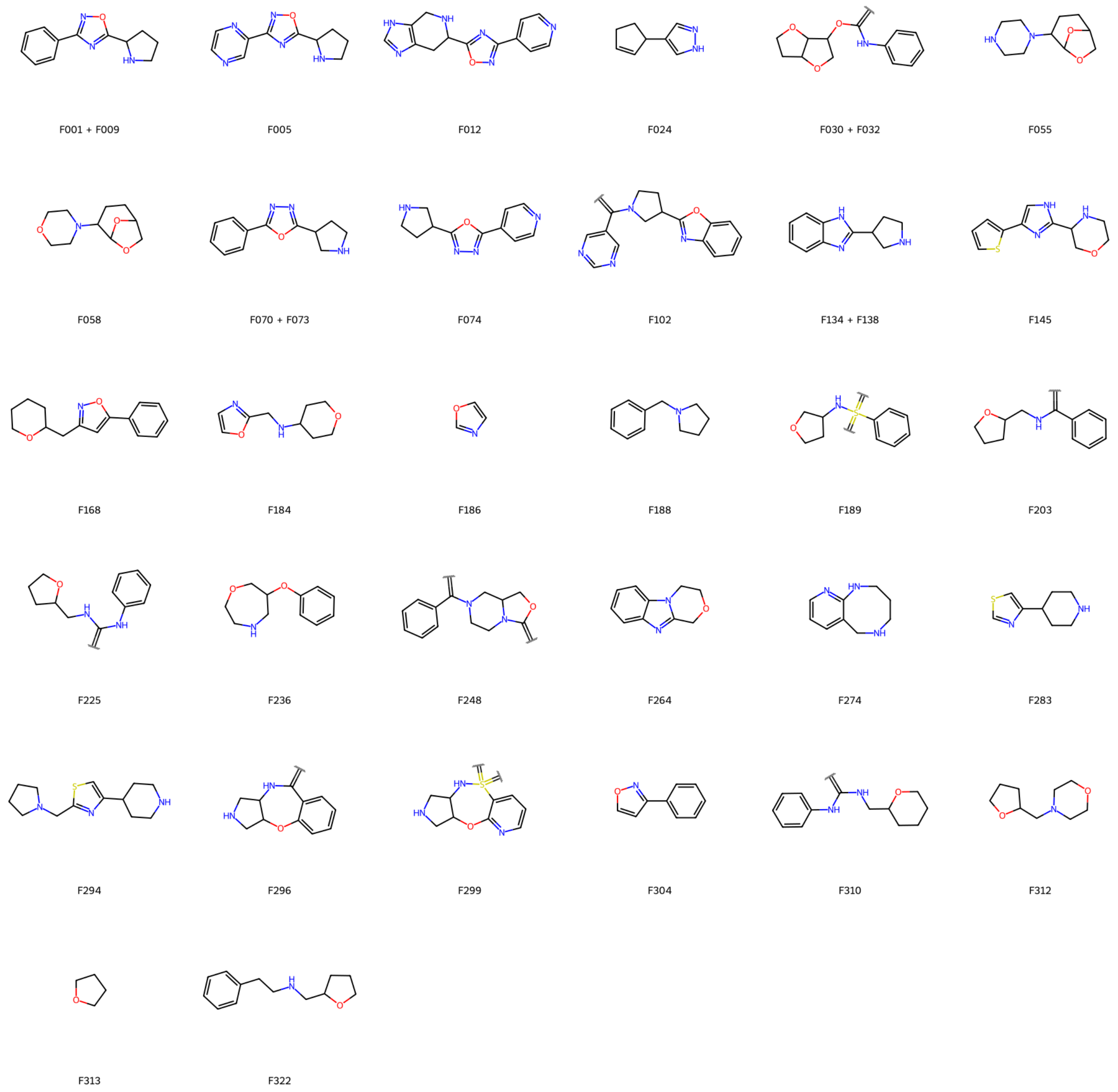

Figure S8: Bemis-Murcko scaffolds of our fragments. None of them is found in the reference datasets.

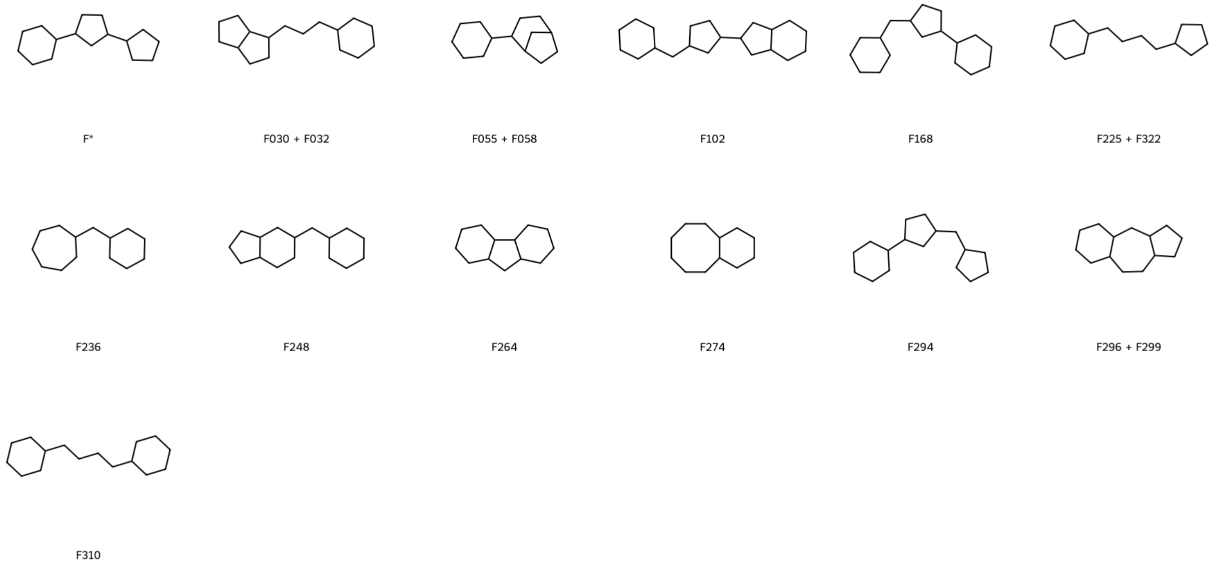

Figure S9: Cyclic skeletons of our fragments, not found in any of the PKA-specific reference datasets. F * = F001 + F005 + F009 + F070 + F073 + F074 + F145.

Chemotypes

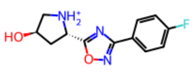

F001

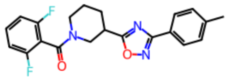

CHEMBL1363915
(ChEMBL Kinases)
0.30

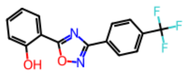

CHEMBL4859206
(ChEMBL Kinases)
0.28

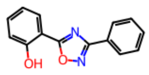

CHEMBL1876217
(ChEMBL Kinases)
0.26

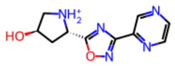

F005

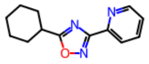

CHEMBL1442840
(ChEMBL Kinases)
0.35

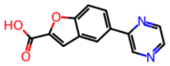

CHEMBL1782507
(ChEMBL Kinases)
0.24

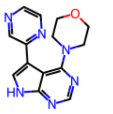

CHEMBL3393442
(ChEMBL Kinases)
0.23

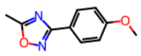

F009

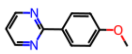

CHEMBL2392358
(ChEMBL Kinases)
0.47

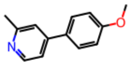

CHEMBL5094650
(ChEMBL Kinases)
0.39

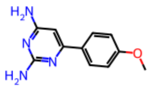

CHEMBL2023701
(ChEMBL Kinases)
0.38

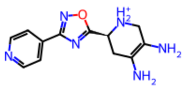

F012

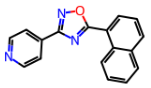

CHEMBL1386396
(ChEMBL Kinases)
0.33

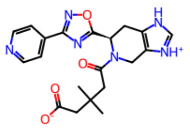

T7W
(PDB)
0.32

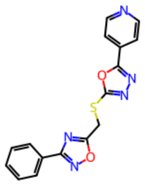

CHEMBL4545000
(ChEMBL Kinases)
0.29

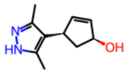

F024

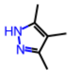

CHEMBL4514142
(ChEMBL Kinases)
0.33

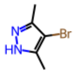

8PQ
(PDB)
0.31

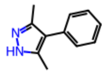

CHEMBL540487
(ChEMBL Kinases)
0.30

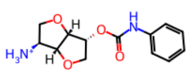

F030

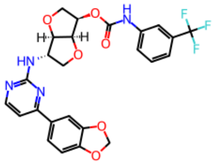

CHEMBL4751957
(ChEMBL Kinases)
0.36

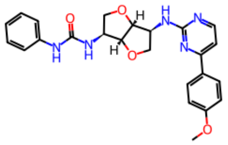

CHEMBL4741384
(ChEMBL Kinases)
0.33

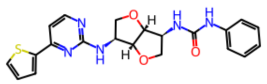

CHEMBL4757545
(ChEMBL Kinases)
0.32

Figure S10: In each row, the fragment structure is shown on the left, along with the three most similar reference molecules from any of the reference datasets to the right. Underneath each reference molecule, the molecule identifier, the reference data set from which it originates and the respective Tanimoto coefficient are printed.

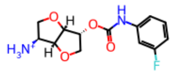

F032

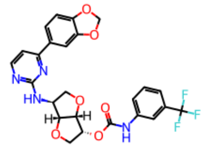

CHEMBL4751957
(ChEMBL Kinases)
0.40

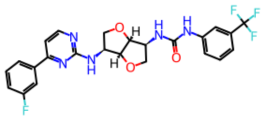

CHEMBL4781499
(ChEMBL Kinases)
0.35

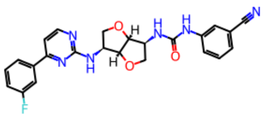

CHEMBL4779572
(ChEMBL Kinases)
0.34

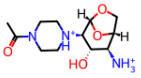

F055

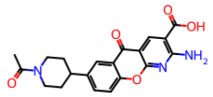

CHEMBL4289613
(ChEMBL Kinases)
0.21

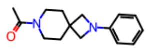

CHEMBL1555190
(ChEMBL Kinases)
0.20

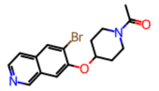

CHEMBL505756
(ChEMBL Kinases)
0.18

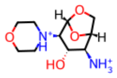

F058

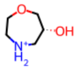

8KB
(PDB)
0.21

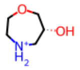

8KB
(PDB)
0.21

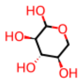

Xylose
(OralDrugs)
0.21

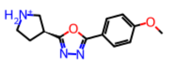

F070

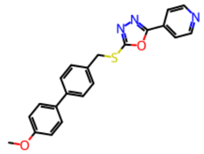

CHEMBL2391131
(ChEMBL Kinases)
0.42

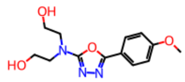

CHEMBL189045
(ChEMBL Kinases)
0.40

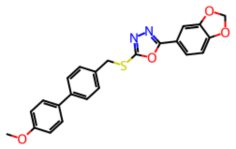

CHEMBL2391119
(ChEMBL Kinases)
0.38

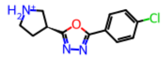

F073

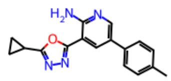

CHEMBL4060747
(ChEMBL Kinases)
0.33

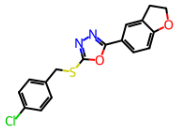

CHEMBL470567
(ChEMBL Kinases)
0.33

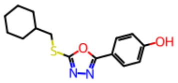

CHEMBL1327547
(ChEMBL Kinases)
0.33

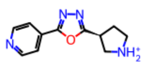

F074

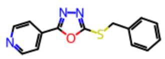

CHEMBL40439
(ChEMBL Kinases)
0.40

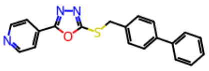

CHEMBL2391099
(ChEMBL Kinases)
0.37

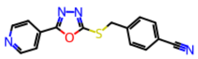

CHEMBL1352577
(ChEMBL Kinases)
0.36

Figure S10: Continued.

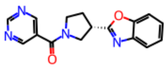

F102

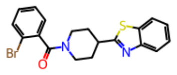

CHEMBL1387577
(ChEMBL Kinases)
0.33

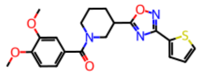

CHEMBL1459223
(ChEMBL Kinases)
0.32

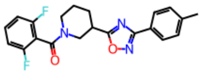

CHEMBL1363915
(ChEMBL Kinases)
0.31

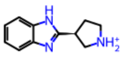

F134

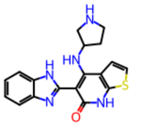

CHEMBL3330134
(ChEMBL Kinases)
0.39

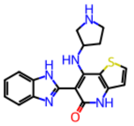

CHEMBL3330237
(ChEMBL Kinases)
0.39

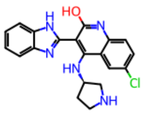

CHEMBL210035
(ChEMBL Kinases)
0.37

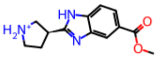

F138

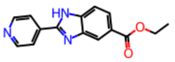

CHEMBL4638916
(ChEMBL Kinases)
0.39

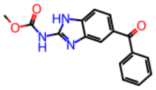

Mebendazole
(OralDrugs)
0.37

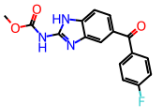

CHEMBL1454946
(ChEMBL Kinases)
0.37

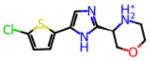

F145

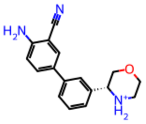

7QI
(PDB)
0.27

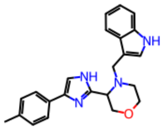

CHEMBL2297166
(ChEMBL Kinases)
0.27

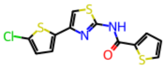

CHEMBL1308898
(ChEMBL Kinases)
0.27

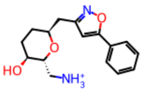

F168

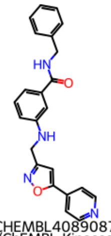

CHEMBL4089087
(ChEMBL Kinases)
0.28

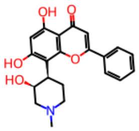

CHEMBL3898028
(ChEMBL Kinases)
0.24

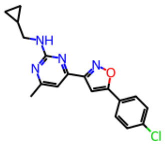

CHEMBL1377267
(ChEMBL Kinases)
0.24

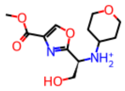

F184

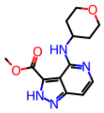

CHEMBL3651815
(ChEMBL Kinases)
0.28

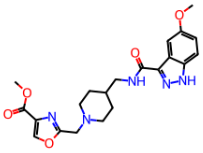

CHEMBL3735500
(ChEMBL Kinases)
0.28

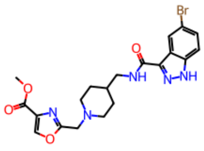

CHEMBL3736476
(ChEMBL Kinases)
0.28

Figure S10: Continued.

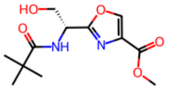

F186

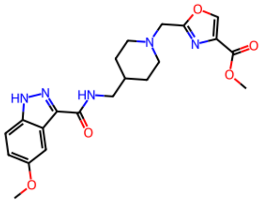

CHEMBL3735500
(ChEMBL Kinases)
0.26

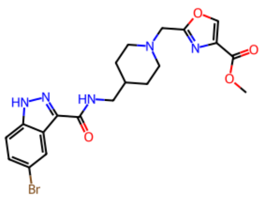

CHEMBL3736476
(ChEMBL Kinases)
0.26

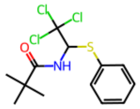

CHEMBL1323500
(ChEMBL Kinases)
0.26

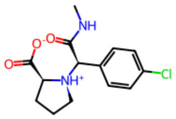

F188

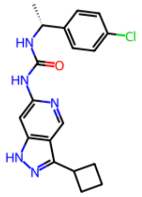

CHEMBL3654781
(ChEMBL Kinases)
0.27

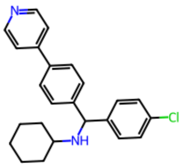

CHEMBL3113137
(ChEMBL Kinases)
0.27

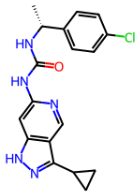

CHEMBL3654780
(ChEMBL Kinases)
0.26

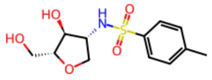

F189

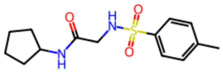

CHEMBL1500529
(ChEMBL Kinases)
0.35

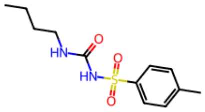

Tolbutamide
(OralDrugs)
0.31

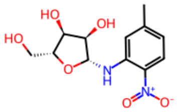

CHEMBL3717069
(ChEMBL Kinases)
0.31

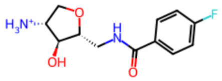

F203

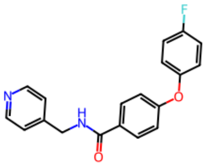

CHEMBL3393291
(ChEMBL Kinases)
0.34

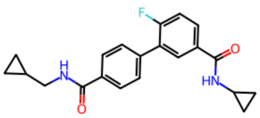

CHEMBL479245
(ChEMBL Kinases)
0.33

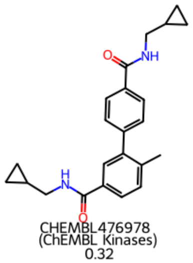

CHEMBL476978
(ChEMBL Kinases)
0.32

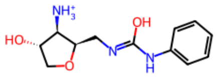

F225

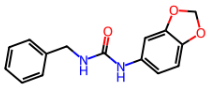

CHEMBL3670690
(ChEMBL Kinases)
0.33

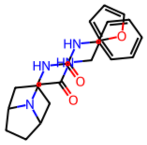

CHEMBL1486429
(ChEMBL Kinases)
0.33

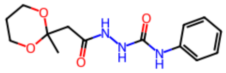

CHEMBL1494943
(ChEMBL Kinases)
0.33

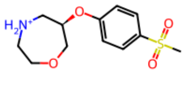

F236

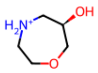

8KB
(PDB)
0.33

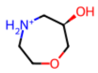

8KB
(PDB)
0.33

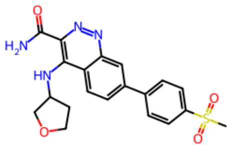

CHEMBL2333134
(ChEMBL Kinases)
0.28

Figure S10: Continued.

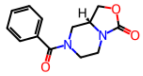

F248

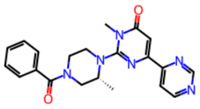

CHEMBL4071773
(ChEMBL Kinases)
0.38

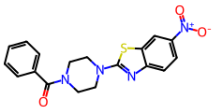

CHEMBL1410177
(ChEMBL Kinases)
0.32

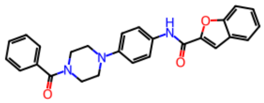

CHEMBL1421442
(ChEMBL Kinases)
0.32

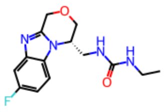

F264

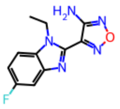

CHEMBL187339
(ChEMBL Kinases)
0.30

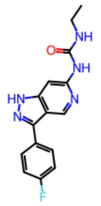

CHEMBL3663058
(ChEMBL Kinases)
0.28

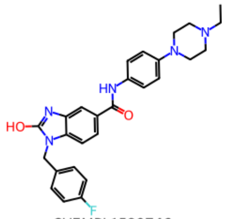

CHEMBL1589746
(ChEMBL Kinases)
0.28

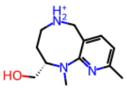

F274

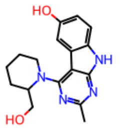

CHEMBL3717442
(ChEMBL Kinases)
0.26

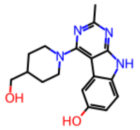

CHEMBL3716916
(ChEMBL Kinases)
0.23

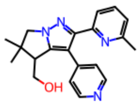

CHEMBL4282287
(ChEMBL Kinases)
0.23

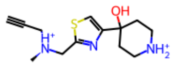

F283

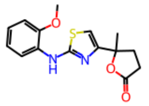

CHEMBL1609453
(ChEMBL Kinases)
0.19

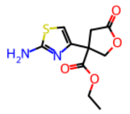

CHEMBL1468526
(ChEMBL Kinases)
0.18

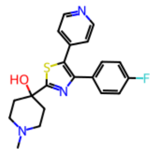

CHEMBL280524
(ChEMBL Kinases)
0.18

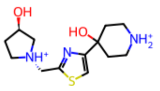

F294

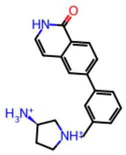

7QW
(PDB)
0.17

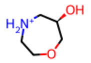

8KB
(PDB)
0.17

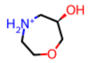

8KB
(PDB)
0.17

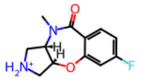

F296

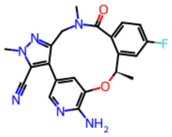

Lorlatinib
(PKIs)
0.28

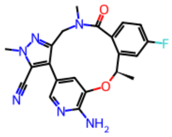

Lorlatinib
(OralDrugs)
0.28

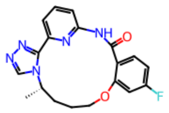

CHEMBL4562269
(ChEMBL Kinases)
0.26

Figure S10: Continued.

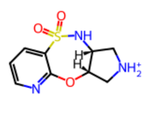

F299

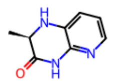

CHEMBL3984982
(ChEMBL Kinases)
0.22

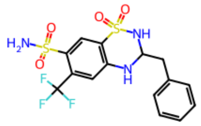

Bendroflumethiazide
(OralDrugs)
0.21

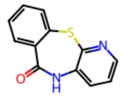

CHEMBL1613269
(ChEMBL Kinases)
0.21

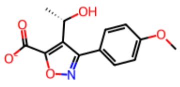

F304

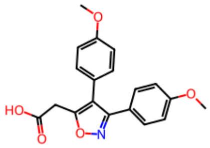

Mofezolac
(OralDrugs)
0.46

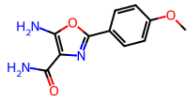

CHEMBL208516
(ChEMBL Kinases)
0.35

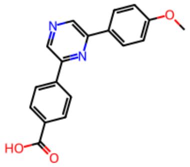

CHEMBL3359882
(ChEMBL Kinases)
0.33

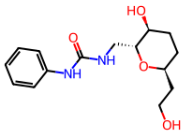

F310

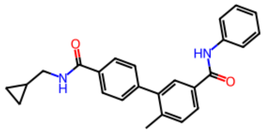

CHEMBL516264
(ChEMBL Kinases)
0.31

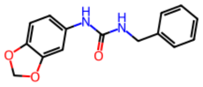

CHEMBL3670690
(ChEMBL Kinases)
0.31

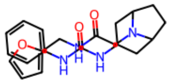

CHEMBL1486429
(ChEMBL Kinases)
0.31

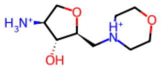

F312

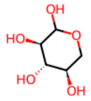

Xylose
(OralDrugs)
0.22

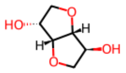

Isosorbide
(OralDrugs)
0.20

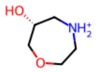

8KB
(PDB)
0.20

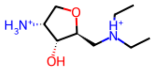

F313

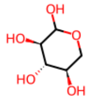

Xylose
(OralDrugs)
0.24

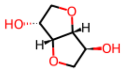

Isosorbide
(OralDrugs)
0.21

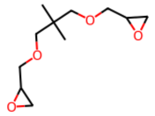

CHEMBL1587512
(ChEMBL Kinases)
0.20

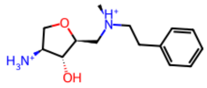

F322

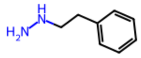

Phenelzine
(OralDrugs)
0.24

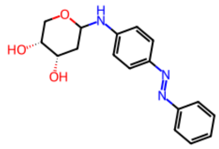

CHEMBL1445664
(ChEMBL Kinases)
0.24

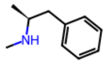

Methamphetamine
(OralDrugs)
0.23

Figure S10: Continued.

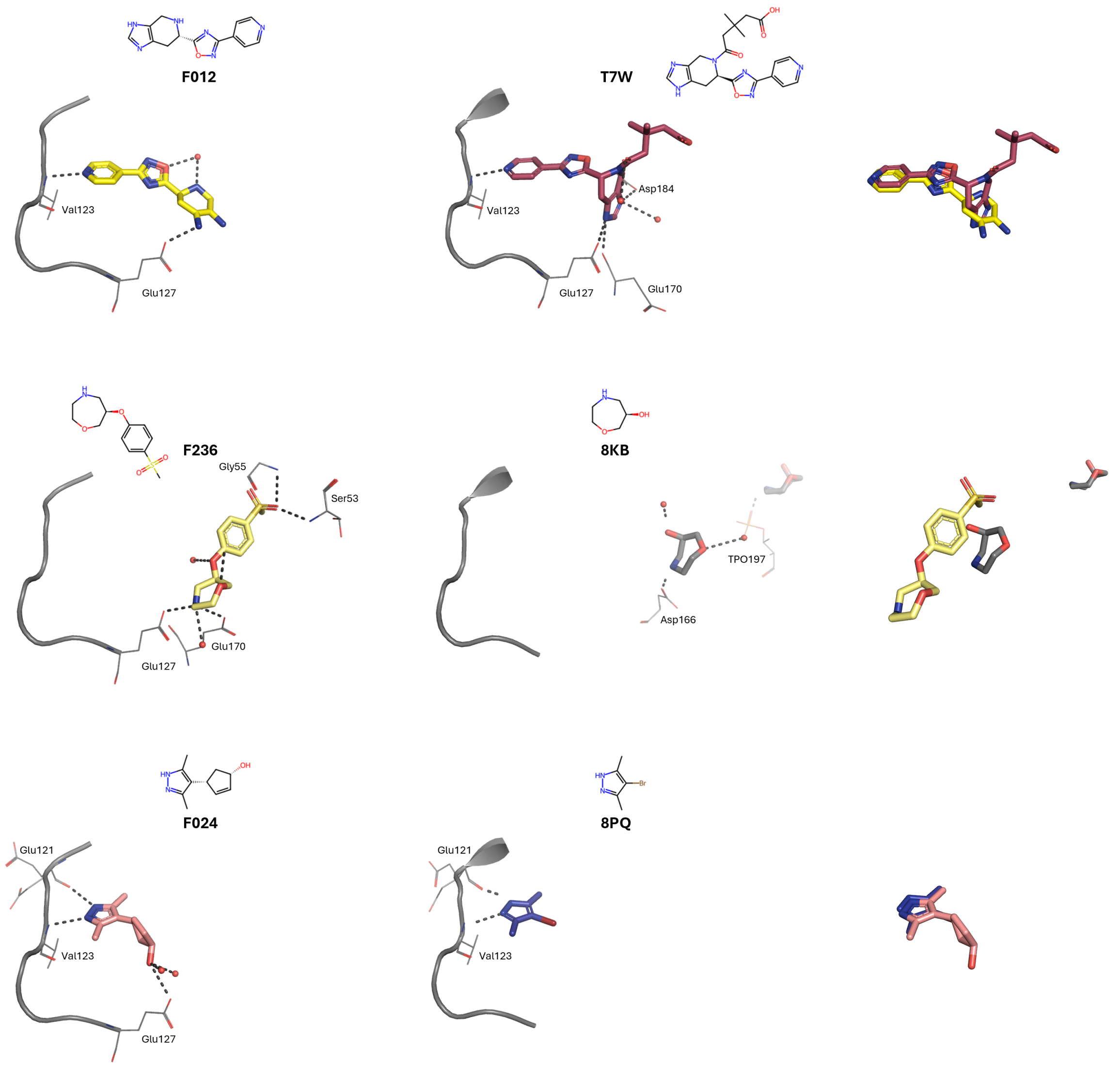

Figure S11: Comparison of Binding Modes for the fragments F012, F236 and F024 and their most similar PKA ligands from the PDB, namely 7TW (PDB-ID: 7BB0, Tanimoto similarity of 0.46 with F012), 8KB (PDB-ID: 5N3G, Tanimoto similarity of 0.33 with F236), 8PQ (PDB-ID: 5N7U, Tanimoto similarity of 0.31 with F024). Fragments are shown as sticks, amino acid residues involved in polar interactions as lines, waters involved in polar interactions as red spheres, and polar interactions as dashed lines. Additionally, the kinase hinge and linker (residues 120−127) are shown in cartoon representation. For clarity, only amino acid residues involved in polar interactions are labeled. On the right, we show the superimposition of the related ligands.

T7W comprises F012 as a substructure, but differs in stereochemistry. Despite this difference, both molecules bind to the same site of the ATP pocket and form a hydrogen bond between the pyridine-N and the backbone amide-NH of the hinge residue Val123, as well as towards Glu127. 8KB is a substructure of F236. Their binding sites are laterally shifted, resulting in different interactions. While the secondary amine of F236 forms a salt bridge with Glu127 and Glu170, 8KB's amino functionality forms a salt bridge to Asp166. 8PQ is an analog of F024 with a bromine substituent in the 4-position of the pyrazole ring, compared to the larger cyclopent-2-en-1-ol moiety in F024. One of the two alternative conformations resolved for 8PQ (B, occupancy = 0.4) closely matches the binding mode of F024, forming hydrogen bonds via the pyrazole nitrogens to the backbone amide-NH of Val123 and the backbone carbonyl-O of Glu121. Moreover, PDB ligand 7QI (PDB-ID: 7PID, Tanimoto similarity of 0.28 with F145) appears in the list of most similar reference molecules, however, 7QI and F145 do not belong to the same chemotype, as they only a small proportion of their structure, and are therefore not compared with regard to their binding modes.

Molecular Descriptors

Table S1: Median values of molecular descriptors. n.d. = not determined.

	MW	HBA	HBD	LogP	NRB	TPSA	NAR	NP-Likeness	NHA	Fsp^3	FC_{stereo}	nSPS	nPBF (*in silico*)	nPBF (protein-bound)
**Binding Site Agnostic**														
**Fragment Library**	250.7	4	2	0.15	4	72	1	-0.41	18	0.55	0.17	27	0.050	n.d.
**Fragment Hits**	253.8	4	2	-0.17	4	73	1	-0.46	18	0.43	0.12	23	0.046	0.041
**PDB**	254.3	3	2	1.39	4	71	2	-0.92	19	0.22	0.00	12	0.036	0.033
**ChEMBL Kinases**	408.5	6	2	3.74	6	87	3	-1.23	29	0.24	0.00	14	0.033	n.d.
**ChEMBL PKA**	371.4	5	2	3.52	6	77	3	-1.12	27	0.22	0.00	15	0.04	n.d.
**Oral Drugs**	349.4	4	2	2.88	7	74	2	-0.47	24	0.38	0.05	16	0.039	n.d.
**PKIs**	467.8	7	2	4.10	8	92	3	-1.27	33	0.28	0.00	15	0.034	n.d.
**COCONUT NPs**	376.4	6	2	2.47	7	86	2	-0.97	27	0.43	0.16	24	n.d.	n.d.
**ATP Site Ligands**														
**Fragment Hits**	250.7	4	2	0.40	4	72	2	-0.87	18	0.42	0.09	22	0.043	0.037
**PDB**	273.4	3	2	1.44	4	72	2	-0.92	20	0.22	0.00	12	0.036	0.032

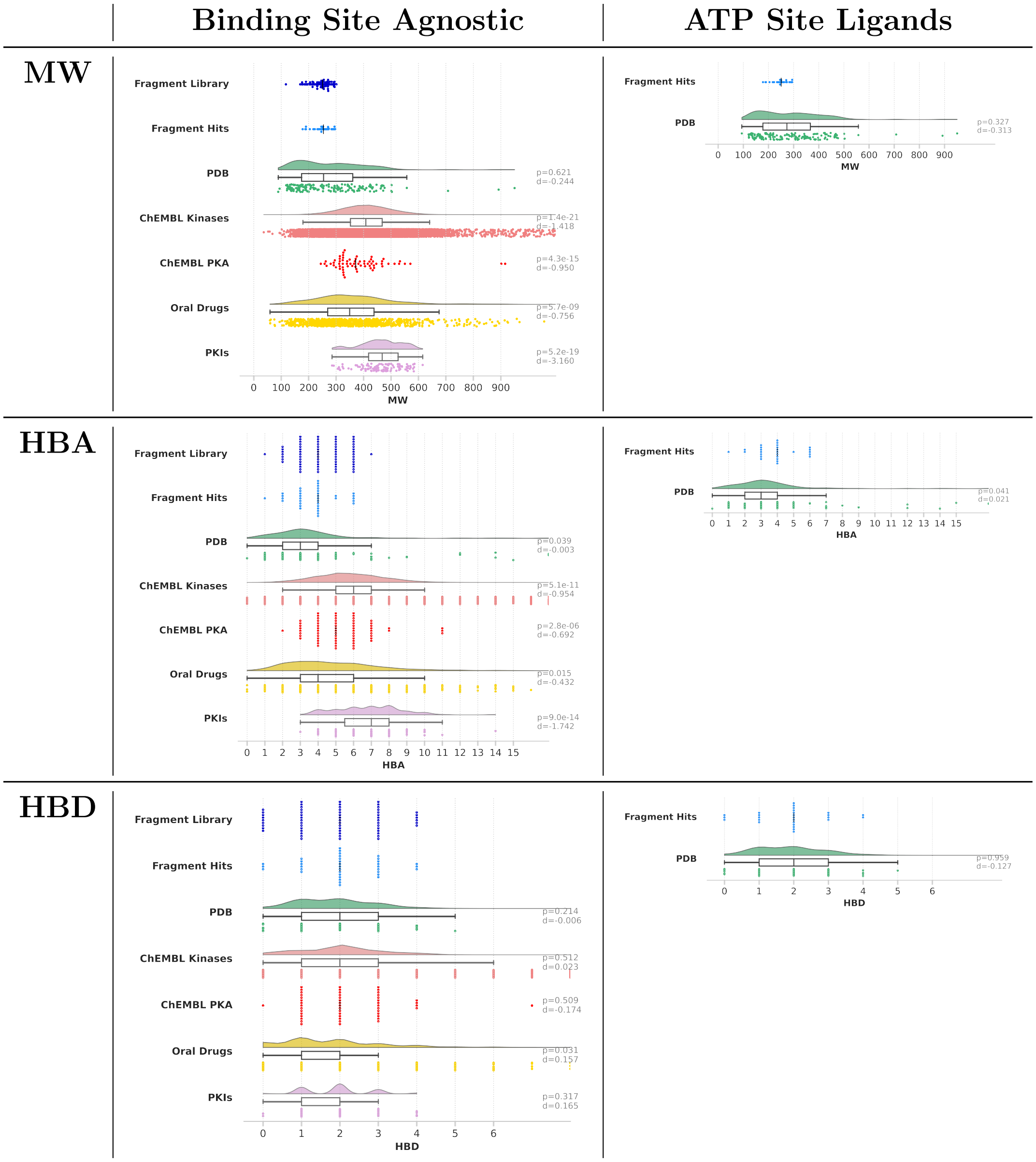

Figure S12: Distributions of descriptor values across the datasets, visualized as rain cloud and swarm plots, respectively, depending on the size of the dataset. In the swarm plots, the median is indicated by a vertical black line. As a guide value for the X-axis limits, the range in which 99% of all the data falls was used. For the subset of ATP-site ligands, we can only compare the datasets with structural information available, namely the fragments and the PDB reference dataset. p-values as obtained from Dunn's statistical test with Bonferroni adjustment, as well as Cohen's d-values, both of which comparing the fragment hits and the reference datasets, are shown on the right. Median values are summarized in Tab. S1.

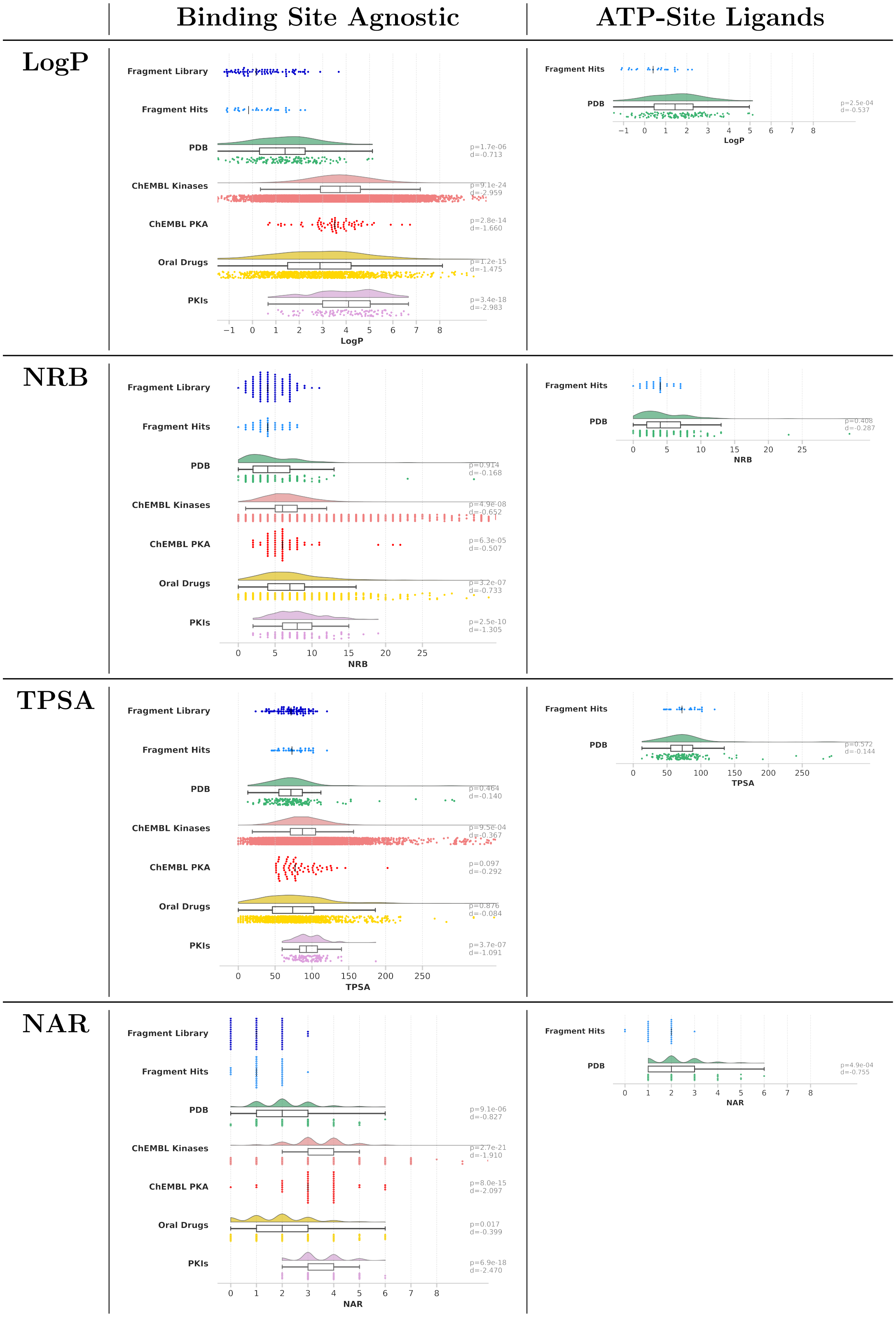

Figure S12: Continued. LogP = calculated logarithm of the n-octanol/water partition coefficient. NRB = Number of rotatable bonds. TPSA = Topological polar surface area. NAR = Number of aromatic rings.

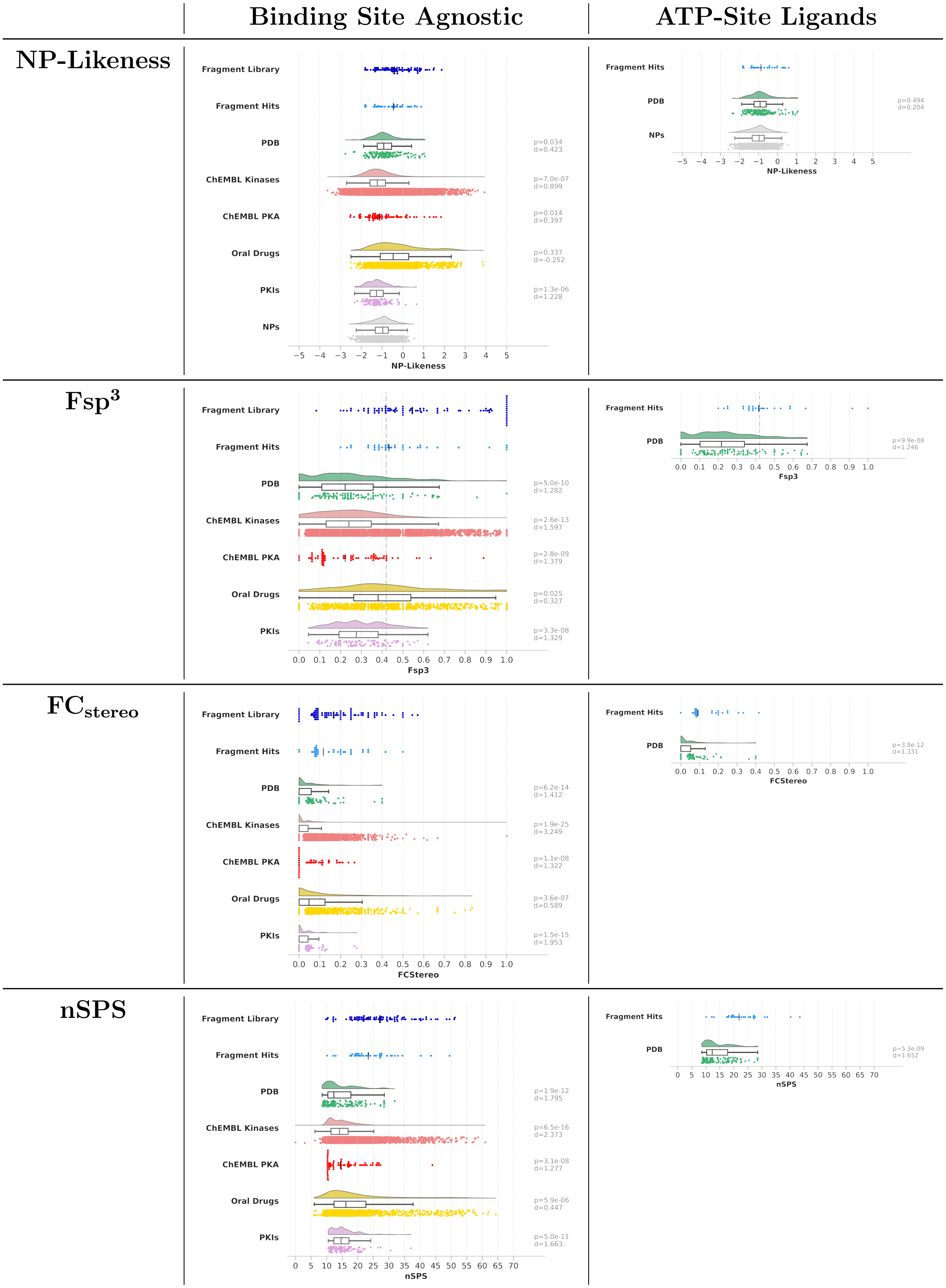

Figure S12: Continued. NP-Likeness = Natural product likeness. Fsp^3 = Fraction of sp^3-hybridized carbons. FC_{stereo} = Fraction of stereogenic carbons. nSPS = Normalized spatial score.

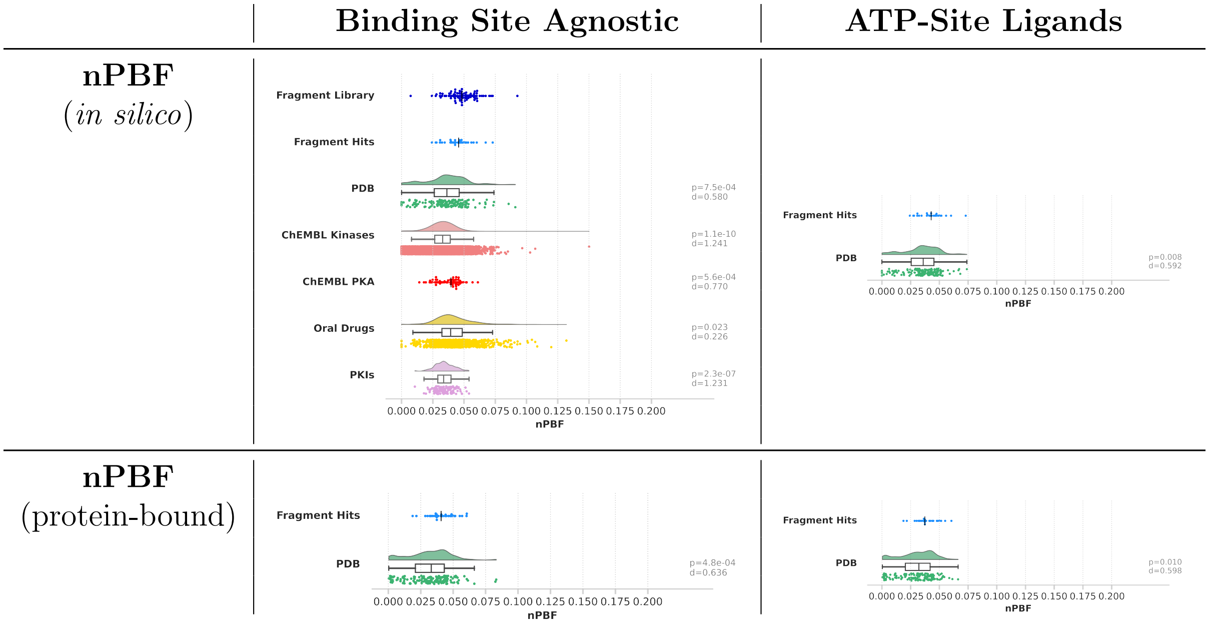

Figure S12: Continued. nPBF = Normalized deviation from the plane of best fit, calculated from *in silico*-generated or the protein-bound ligand conformations.

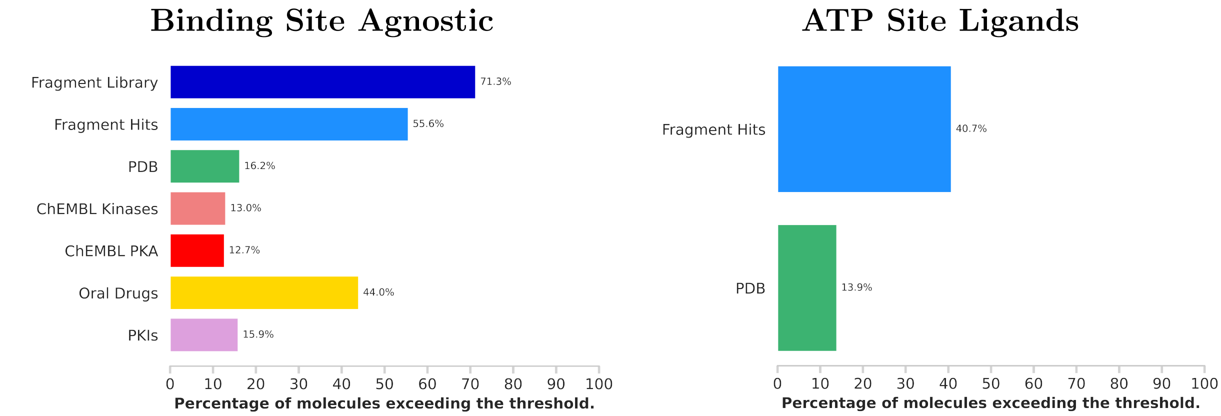

Figure S13: Percentage of molecules exceeding the threshold of $Fsp^3 \geq 0.42$ defined by Kombo *et al.*.[64]

Table S2: Molecules exhibiting the highest/lowest descriptor value per dataset. '...' indicates that more than 5 molecules possess the exact same value. n.d. = not determined. A color code similar to the one used in Fig. 1 is used: Fragments that bind only in the ATP pocket are highlighted in green; fragments that bind only at one or more peripheral site(s) are highlighted in blue; fragments binding in the ATP pocket and at least one additional peripheral site are printed in black.

Dataset	NP Likeness	Fsp^3	FC_{stereo}	nSPS	nPBF (*in silico*)	nPBF (protein-bound)
**Molecules exhibiting the highest descriptor value**						
	F313 (+0.88)	F058/F312/F313 (1.00)	F058 (0.50)	F058 (50)	F312 (0.073)	F312 (0.061)
	F058 (+0.68)	F055 (0.92)	F055 (0.42)	F055 (44)	F313 (0.067)	F058 (0.060)
**Fragment Hits**	F312 (+0.58)	F294 (0.77)	F312/F313 (0.33)	F312 (40)	F024 (0.060)	F313 (0.057)
	F322 (+0.56)	F184 (0.67)	F030/F032 (0.31)	F313 (34)	F189 (0.058)	F264 (0.055)
	F055 (+0.40)	F283 (0.62)	F225 (0.25)	F030 (33)	F005 (0.056)	F024 (0.052)
**Molecules exhibiting the lowest descriptor value**						
	F264 (–1.82)	F009 (0.20)	F009/F283 (0.00)	F009 (10)	F009 (0.024)	F012 (0.019)
	F009 (–1.81)	F304 (0.23)	F102 (0.06)	F304 (12)	F138 (0.027)	F009 (0.022)
**Fragment Hits**	F102 (–1.78)	F102/F012 (0.25)	F264 (0.07)	F186 (13)	F070 (0.028)	F304 (0.029)
	F001 (–1.39)	F001/F073 (0.33)	..... (0.08)	F264 (18)	F073 (0.031)	F138 (0.030)
	F005 (–1.35)	F074 (0.36)	..... (0.08)	F184 (18)	F102 (0.031)	F001 (0.032)

Dataset	NP Likeness	Fsp^3	FC_{stereo}	nSPS	nPBF (*in silico*)	nPBF (protein-bound)
**Molecules exhibiting the highest descriptor value**						
**PDB**	Staurosporine [STU] (+1.06)	8KB (1.00)	ATP/ADP/AMP/ANP (0.40)	8KB (32)	8KB (0.091)	8KB/152 (0.083)
**PKIs**	Midostaurin (+0.66)	Gilteritinib (0.62)	Peficitinib (0.28)	Peficitinib (37)	Repotrectinib (0.054)	n.d.
**ChEMBL PKA**	CHEMBL3099612 (+1.84)	Sphingosine (0.89)	CHEMBL4080906 (0.27)	CHEMBL4080906 (44)	CHEMBL148333 (0.061)	n.d.
**ChEMBL Kinases**	CHEMBL1356390 (+3.93)	..... (1.00)	CHEMBL23552 (1.00)	CHEMBL1596335 (61)	Propionaldehyde (0.150)	n.d.
**Oral Drugs**	Artenimol (+3.88)	..... (1.00)	Kanamycin (0.83)	Memantine (64)	Cysteamine (0.132)	n.d.

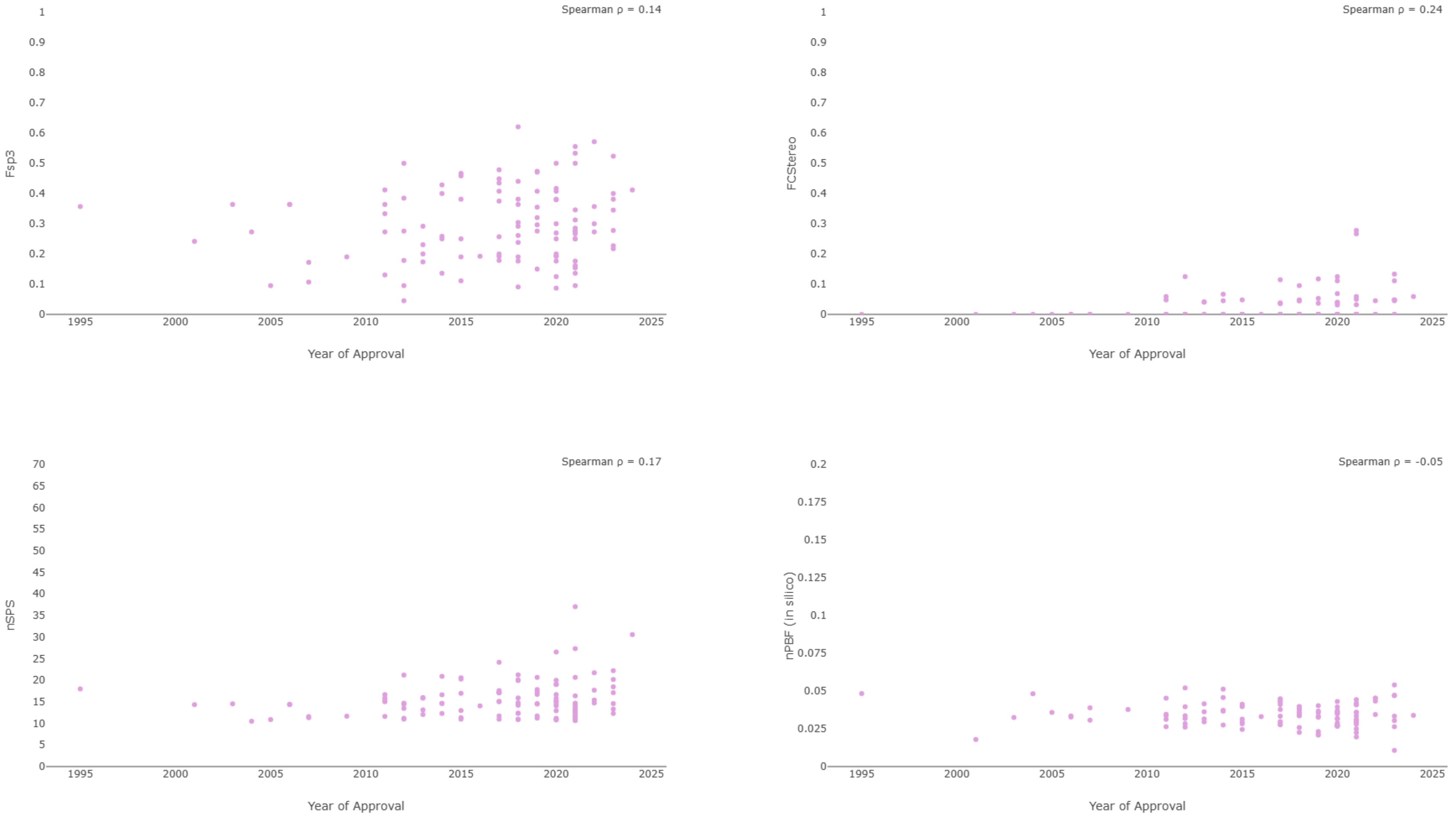

Figure S14: Correlation between the PKI's approval year and selected descriptor values.
